# One-Pot Synthesis of Cellulose/MXene/PVA Foam for Efficient Methylene Blue Removal

**DOI:** 10.3390/molecules27134243

**Published:** 2022-06-30

**Authors:** Weisong Zhao, Hong Chi, Shiyun Zhang, Xue Zhang, Tianduo Li

**Affiliations:** Provincial Key Laboratory of Molecular Engineering, School of Chemistry of Chemical Engineering, Qilu University of Technology (Shandong Academy of Sciences), Jinan 250353, China; wsmail2022@163.com (W.Z.); whitesnow0613@163.com (S.Z.); chihqut@163.com (X.Z.)

**Keywords:** foam, Ti_3_C_2_T_x_ MXene, polyvinyl alcohol, cellulose nanocrystals, methylene blue, adsorption

## Abstract

Ti_3_C_2_T_x_ MXene has attracted considerable interest as a new emerging two-dimensional material for environmental remediation due to its high adsorption capacity. However, its use is greatly limited by its poor mechanical properties, low processability and recyclability, and the low dispersity of such powder materials. In this work, a porous adsorbent (C–CMP) containing cellulose nanocrystals (CNC), Ti_3_C_2_T_x_ MXene and polyvinyl alcohol (PVA) was prepared by a simple and environmentally-friendly foaming method. Glutaraldehyde was used as crosslinker to improve the mechanical properties and boost the adsorption efficiency of methylene blue (MB) molecules. Fourier transform infrared (FT–IR), elemental analysis (EDX) and thermogravimetric analysis (TGA) further confirmed that the preparation of the C–CMP foam and cross-linking reaction were successful. Scanning electron microscope (SEM) indicated that the macropores were distributed homogeneously. The adsorption experiment showed that maximum adsorption capacity of MB can reach 239.92 mg·g^−1^ which was much higher than anionic dye (methyl orange, 45.25 mg·g^−1^). The adsorption behavior fitted well with the Langmuir isotherm and pseudo-second-order kinetic models. Thermodynamic analysis indicated that the adsorption process was spontaneous and endothermic. Based on FT–IR, EDX and X-ray photoelectron spectroscopy (XPS) analysis, the adsorption mechanism between C–CMP and MB molecules was attributed to electrostatic interaction.

## 1. Introduction

It is estimated that more than 100,000 different types and 7 × 10^5^ tons of commercial dyes are produced annually in the world [1,2,3]. Due to the complex molecular structure and chemical resistance of dyes, dye pollution is still a major concern in regard to water pollution worldwide [4]. Methylene blue (MB) is widely used for coloring paper, cotton, silk, wool and hair [5,6]. Unfortunately, methylene blue is difficult to degrade due to its benzene ring structure, and therefore it accumulates in drainage systems. Even if the concentration of MB is as low as 1 mg L^−1^, the transparency and dissolved oxygen of water will be severely affected [7].

Therefore, developing effective methods to remove MB from wastewater is critical to maintaining environmental sustainability and public health [8,9,10,11]. Foam adsorbents have been well developed due to their easy processing and low energy consumption [12,13]. For example, Ren et al. prepared a novel composite foam (CMCTS-PUF-s) by immobilizing carboxymethyl chitosan (CMCTS) on polyurethane foam (PUF) in which the amino group in CMCTS reacted with the isocyanate group in polyurethane prepolymer [14]. The adsorption capacity of the foam (CMCTS-PUF-5) reached 118.2 mg·g^−1^ at a 5% CMCTS loading and the removal rate of MB reached 97.1%. In the same year, Zhan et al. fabricated nontoxic, environmentally-friendly, and multifunctional super-wetted graphene oxide (GO)/g–C_3_N_4_/TiO_2_ (GCT) foam by hydrothermal treatment and freeze–drying [15]. The prepared GCT foam can simultaneously separate oil–water mixture and adsorb water-soluble organic dyes, and both the separation efficiency and adsorption efficiency were higher than 98%.

A thriving class of two-dimensional (2D) transition metal carbides, nitrides and carbonitrides, MXenes are produced by selective etching of A elements from MAX phases, where A is a group IIIA to VIA element. The typical formula is M_n+1_X_n_T_x_, where M is a transition metal, X represents C and/or N, *n* = 1–3, and T_x_ are functional groups on the surface (OH, −O, or −F). MXenes can also be used as an ideal reinforcing nanofillers for foam adsorbents due to their excellent flexibility, chemical tunability, and hydrophilic surfaces, etc. Only a small amount of MXene was reported to significantly improve the mechanical properties of the adsorbent [16,17,18]. However, utilization of MXenes as an adsorbent is greatly limited by the poor mechanical properties, and low processability and recyclability.

Herein, with the aim of sewage purification, especially dye wastewater treatment, a macroporous cellulose nanocrystals (CNC)/MXene/polyvinyl alcohol (PVA) (C–CMP) foam was prepared by a simple one–pot method for MB removal from wastewater. PVA was employed as a polymer matrix due to its cost–effectiveness and hydrophilicity. The large amount of hydroxyl groups can also promote intermolecular and intramolecular hydrogen bonds to boost the mechanical performance of the resulting materials [19]. Due to its good biocompatibility and mechanical strength, PVA is widely used in environmental remediation.

To improve the mechanical and adsorption capacity of the composite as well, CNC with diameters between 2 and 20 nm and lengths of up to tens of micrometers are preferable. CNC is an important source of nanoscale fillers that have loading–carry capacity [20]. The use of CNC in the preparation of adsorbent materials has been investigated not only because of their interesting physical and chemical properties, but also their inherent reproducibility, sustainability, and abundance. Glutaraldehyde (GLA) was used as a cross–linking agent to improve the stability of the foam during the adsorption process. The as–prepared foams were characterized using Fourier transform infrared (FT–IR), elemental analysis (EDX), scanning electron microscopy (SEM) and thermogravimetric analysis (TGA). In addition, the effects of MXene content, pH value, initial MB concentration, MB adsorption time and temperature on the adsorption performance of C–CMP for MB were investigated, and the adsorption process was analyzed in detail using adsorption isotherm, kinetic and thermodynamic models. We expected that the composite foam can play an important role in dye wastewater treatment, and realize the sustainable development of water resources.

## 2. Results and Discussion

### 2.1. Characterization of C–CMP Composite Foam

**Figure 1:**
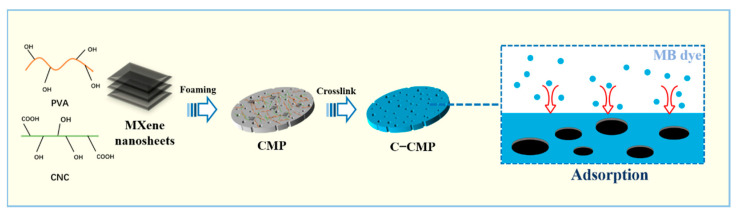
Preparation process of cross–linked cellulose nanocrystals (CNC)/MXene/polyvinyl alcohol (PVA) (C–CMP) composite foam.

**Figure 2:**
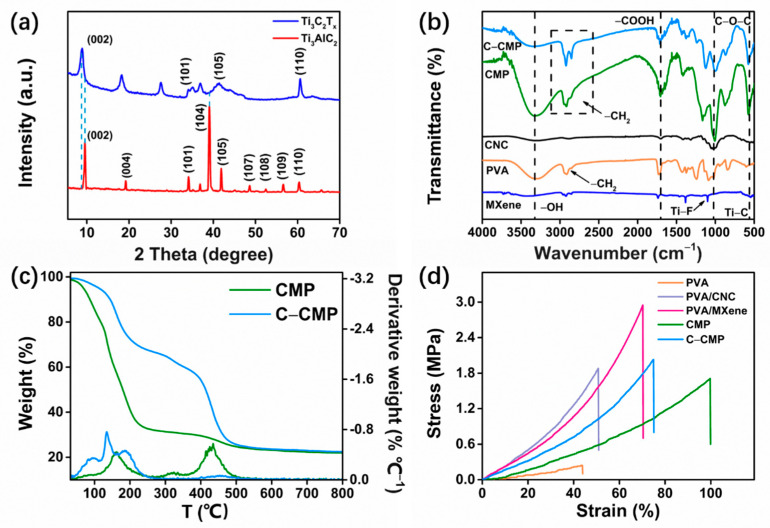
(**a**) X-ray diffraction (XRD) spectra of Ti_3_AlC_2_ and Ti_3_C_2_T_x_. (**b**) Fourier transform infrared (FT–IR) spectra of Ti_3_C_2_T_x_ MXene, polyvinyl alcohol (PVA), cellulose nanocrystals (CNC), uncross–linked CNC/MXene/PVA (CMP) composite foam and C–CMP. (**c**) Thermogravimetry analysis/derivative thermogravimetry (TGA/DTG) curves of CMP and C–CMP composite foams. (**d**) Stress–strain curves of PVA, PVA/CNC, PVA/MXene, CMP and C–CMP foams.

The overall preparation process of C–CMP is shown in Figure 1. The X-ray diffraction (XRD) in Figure 2a shows that after the treatment of Ti_3_AlC_2_ with HF, the characteristic peak at ~39° (104) completely disappears, confirming that the Al layer is successfully removed. Furthermore, the significant decrease in the (101) and (105) peak intensities of Ti_3_AlC_2_ confirms the transition from Ti_3_AlC_2_ to Ti_3_C_2_T_x_. In addition, the (002) diffraction peak of Ti_3_AlC_2_ shifts from 9.52° to 8.92°, which also indicates an increase in the interlayer spacing. The Ti_3_C_2_T_x_ obtained after etching shows a good accordion–like multilayer structure with a relatively smooth surface composed of many nanoflakes with a lateral size > 2 μm and a thickness of 30–100 nm (Figure S1).

FT–IR spectroscopy was used to study the characteristic functional groups of Ti_3_C_2_T_x_ MXene, PVA, CNC, CMP and C–CMP as shown in Figure 2b. The infrared spectrum of Ti_3_C_2_T_x_ shows an absorption peak at 3429 cm^−1^, which is attributed to the stretching vibration peak of the hydroxyl group (–OH). The peaks at 570 and 1100 cm^−1^ correspond to the vibrations of Ti–C and Ti–F, respectively. PVA exhibits a strong −CH_2_ stretching vibration band at 2912 cm^−1^ and at 3301 cm^−1^, which is attributed to the stretching vibration of −OH in PVA. CNC exhibits characteristic absorption peaks at 3301 cm^−1^, 1702 cm^−1^ and 1026 cm^−1^, corresponding to the stretching vibration bands of −OH, −COOH and C–O–C, respectively. There are Ti–C, C–O–C, −COOH, C–H (methylene) and −OH vibrations in the FT–IR spectra of CMP and C–CMP, indicating that composite foams were successfully prepared and contain amounts of Ti_3_C_2_T_x_ MXene, PVA and CNC. EDX was used to evaluate the content of organic elements in different samples, as shown in Table S1. It is worth noting that after the addition of GLA reagent, the C content in C–CMP reaches 52.91 wt%, which is higher than that of CMP (48.23 wt%). Furthermore, the improvement of the total ratio (94.00 wt%) in C–CMP also confirms the success of the cross–linking reaction.

TGA and derivative thermogravimetry (DTG) curves of CMP and C–CMP composite foams are shown in Figure 2c. A three–step weight loss is observed in CMP. The first step at temperatures up to 100 °C, involves the release of absorbed and bound water present in the foam matrix, while the second degradation occurs over 300 °C, resulting in a nearly 69% weight loss, which involves the decomposition of oxygen–containing groups and polymer backbone. The third step might involve the decomposition of residual carbon from CNC, MXene and PVA [21]. The addition of GLA causes the most significant improvement in the thermal stability of C–CMP, where the initial decomposition temperature shifts to 155 °C. Furthermore, chemical cross–linking makes the decomposition of the C–CMP composite foam shift to 560 °C, most likely due to the cross–linking of the polymer backbone with CNC and Ti_3_C_2_T_x_ MXene [21,22,23,24].

As a polymer matrix, PVA exhibits excellent elastic behavior. As shown in Figure 2d and Table 1, CNC and MXene as fillers significantly affect the tensile strength and elongation at break of the foams. The elongation at break and tensile strength of PVA were only 43.96% and 0.24 Mpa, respectively. After adding CNC and MXene, the elongation at break and tensile strength increased significantly, reaching 50.96%, 70.44% and 1.88 Mpa, 2.95 Mpa, respectively. Notably, due to the synergy between CNC and MXene, the largest elongation at break was observed in CMP, which improved by 127.25% compared to PVA. After adding GLA agent, C–CMP obtained greater tensile strength than CMP, which was attributed to the increase in hardness after cross–linking.

**Figure 3:**
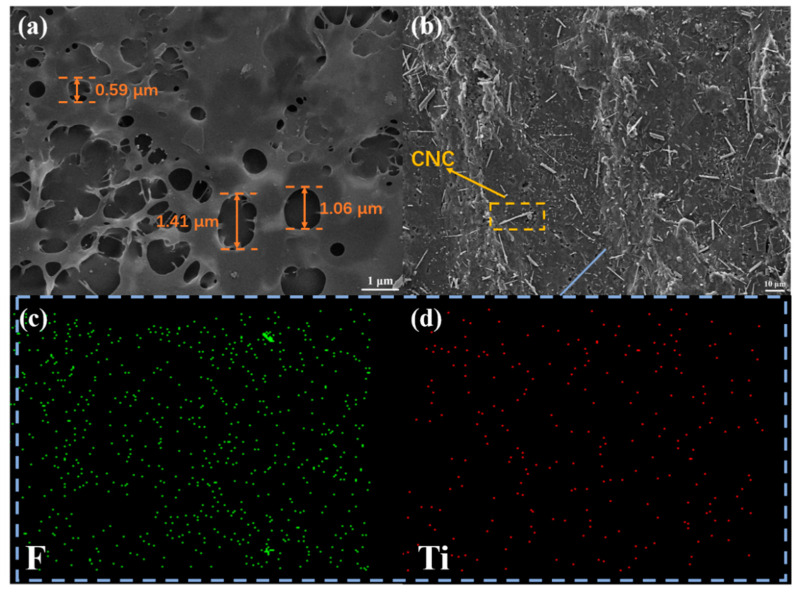
(**a**,**b**) Scanning electron microscopy (SEM) images of C–CMP composite foam. (**c**,**d**) Elemental mapping images of C–CMP showing the presence of F and Ti.

In order to observe the prepared foam more intuitively, we studied the microstructure of C–CMP through SEM. The C–CMP has a large quantity of bubble–like micropores with a relatively uniform and dense distribution as shown in Figure 3a, indicating the success of the foaming process. It can be seen from Figure 3b that there are also some crystal structures on the whole foam, which has been washed with ethanol several times. In addition, F and Ti elements are uniformly distributed on the surface of C–CMP after preparation (Figure 3c,d). Therefore, we could infer that the CNC (100–500 nm in length) and Ti_3_C_2_T_x_ MXene were evenly distributed and anchored on the surface of PVA after the addition of GLA.

### 2.2. Adsorption Performance Study of MB

**Figure 4:**
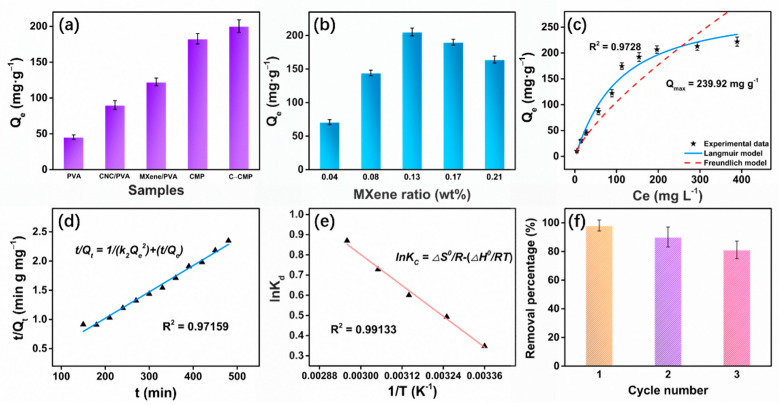
(**a**) Equilibrium adsorption capacity of different samples in MB adsorption. (**b**) Effects of Ti_3_C_2_T_x_ MXene feeding ratios on MB adsorption capacity. (**c**) Langmuir and Freundlich isotherms of the adsorption of MB by C–CMP. (**d**) Pseudo–second–order kinetic model of MB adsorption by C–CMP. (**e**) The Van’t Hoff equation model for MB removal over C–CMP. (**f**) Recyclability test of C–CMP. Initial concentration of MB was (**a**,**b**) 250 mg L^−1^, (**d**,**e**) 250 mg L^−1^, (**f**) 50 mg L^−1^.

The equilibrium adsorption capacity of various foams for MB were compared (Figure 4a). Similar to the tensile test, 2D–layered Ti_3_C_2_T_x_ MXene was significantly better than CNC for improving MB removal. Considering the synergistic effect between CNC and Ti_3_C_2_T_x_ MXene, and the large–scale increase in −OH and −COOH functional groups on the composite foam, the adsorption capacity of C–CMP reached 200.28 mg·g^−1^, the largest among the samples, which was 341.53% higher than that of the unmodified PVA (45.36 mg·g^−1^). In order to optimize the ratio of Ti_3_C_2_T_x_, PVA and CNC were then mixed with MXene in various weight ratios as shown in Figure 4b. The MB adsorption amount of C–CMP containing different ratios of Ti_3_C_2_T_x_ MXene was investigated, corresponding to 0.04 wt%, 0.08 wt%, 0.13 wt%, 0.17 wt% and 0.21 wt%. It was found that when the MXene content reached 0.13 wt%, the adsorption of MB reached a maximum value of 205.34 mg·g^−1^. When the ratio was further increased to 0.21 wt%, the adsorption amount decreased by 20.08%. Excessive Ti_3_C_2_T_x_ will inevitably lead to stacking of its 2D lamellar structure, disorder of the foam space and a reduction in surface–active sites, thus hindering further improvement in the MB adsorption performance. Therefore, all subsequent adsorption experiments were performed with a MXene ratio of 0.13 wt% (hereafter referred to as C–CMP).

The corresponding surface charge of adsorbents in aqueous solution varies significantly with pH value [25]. Therefore, MB adsorption experiments were performed at different pH values (2.0–10.0) of the solution. Figure S2 shows that the adsorption capacity of C–CMP foam increased from 50.35 to 206.58 mg·g^−1^ with the gradual increase in pH. We speculate that the variation in the adsorption amount with pH value can be explained by the surface charge of C–CMP. Due to the presence of a large amount of H^+^ in the solution under acidic conditions (pH = 2.0~4.0), protonation of the adsorption sites (–COOH and −OH) leads to electrostatic repulsion between C–CMP foam and cationic MB, resulting in lower adsorption capacity (50.35 mg·g^−1^ to 112.32 mg·g^−1^). In contrast, when the pH of the solution is higher than 6.0 or the solution is under alkaline conditions, the negative charge (OH^−^) around the composite foam increases significantly, which attracts the cationic MB dye towards the adsorbent. In addition, −OH and −COOH of C–CMP are ionized and interact with MB dye molecules via the strong electrostatic interaction, which favors the higher removal ability of MB at higher pH. Since the adsorption amount of C–CMP gradually reached saturation (185.21 ± 5.67 mg·g^−1^) at pH 6.0, the pH value was fixed at 6.0 in the subsequent experiments.

In order to evaluate the effect of the initial MB concentration on C–CMP equilibrium adsorption, MB solution with different initial concentrations (10, 30, 50, 100, 150, 200, 250, 300, 400 and 500 mg·L^−1^) was prepared for batch adsorption experiments. As shown in Figure S3, the adsorption capacity of the foam increased rapidly when the initial MB concentration was in a low range (10–200 mg·L^−1^). After that, with the continuous increase in concentration, the amount of MB adsorption reached saturation (192.15 ± 8.50 mg·g^−1^) at the concentration of 250 mg·L^−1^. Due to limited adsorption sites in the foam, the adsorption amount of C–CMP will only be maintained within a specific range even if the solution (300–500 mg·L^−1^) contains excess MB molecules. In addition, we also investigated the removal ability of C–CMP for the anionic dye methyl orange (MO) in aqueous solution under the same experimental condition (Figure S4), and found that its adsorption capacity (45.25 mg·g^−1^) was much lower than that of MB. This is mainly due to the electrostatic repulsion between MO molecules and C–CMP foam. Therefore, we believe that C–CMP can be used as an ideal adsorbent material for the efficient removal of cationic dyes from industrial wastewater, contributing to environmental remediation through electrostatic interaction.

The Langmuir and Freundlich adsorption isotherm models describe the adsorption process on homogeneous (monolayer) and heterogeneous (multilayer) surfaces, respectively [26,27,28]. The two models can be expressed as:

(1) $$Q_{e}=\frac{Q_{m}K_{L}C_{e}}{{1+K}_{L}C_{e}}$$

(2) $$Q_{e}{=K}_{F}C_{e}^{\frac{1}{n}}$$

where *K_L_*, *Q_m_*, *K_F_*, and *n* are defined as the Langmuir constant (L/mg), theoretical maximum adsorption capacity (mg·g^−1^), Freundlich constant (L/mg) and adsorption strength, respectively.

The obtained adsorption parameters are shown in Figure 4c and Table S2. Compared with the Freundlich isotherm model, R^2^ of the Langmuir isotherm model is 0.9728, which is more suitable for describing adsorption process of the foam, indicating that MB is adsorbed on the active sites with equivalent energy and there is no interaction between the MB molecules. The theoretical maximum adsorption capacity is 239.92 mg·g^−1^, which is consistent with the experimental data. The Freundlich constant (*n*–heterogeneity factor) obtained by fitting >1 indicates that C–CMP is beneficial to the adsorption of MB dye [29]. It can be inferred that the presence of chemical adsorption makes the adsorption rate faster and the time to reach adsorption equilibrium is shorter.

The contact time of adsorbates is another important factor affecting the adsorption equilibrium in solution systems [30]. Figure S5 shows the dependence of C–CMP adsorption of MB on contact time. In the initial stage, MB molecules were adsorbed quickly onto the foam surface with the adsorption capacity reaching up to 198.16 mg·g^−1^ within 3 h, which is related to the abundant active sites exposed on the surface of C–CMP. With an increase in the subsequent contact time, the MB molecules are difficult to capture due to the lack of active adsorption sites on the foam surface, and the adsorption capacity gradually reaches equilibrium.

The adsorption kinetics were studied using pseudo–first–order and pseudo–second–order kinetic models [31,32]. The equations for the two models are as follows:

(3) $$Q_{t}\;{=\;Q}_{e}\left(1\;-\;exp\left(–k_{1}t\right)\right)$$

(4) $$\frac{t}{Q_{t}}\;=\;[\frac{1}{k_{2}Q_{e}^{2}}]\;+\;\frac{1}{Q_{e}}t$$

where *k*_1_ (min^−1^) and *k*_2_ (g mg^−1^ min^−1^) are the rate constants of the two kinetic models, and *Q_t_* (mg·g^−1^) is the adsorption amount of MB at time *t*. The fitting results of the pseudo–first–order and pseudo–second–order kinetic models are summarized in Table S3. Accordingly, the pseudo–second–order kinetic model is more appropriate for describing the adsorption of MB by C–CMP (*R*^2^ = 0.97159), as shown in Figure 4d, indicating that the adsorption process is mainly controlled by chemical adsorption. At the concentration of 250 mg L^−1^, the MB adsorption amount obtained by fitting was 221.24 mg·g^−1^, which is closer to the experimental data. The structural design of the porous foam and presence of large amounts of −OH and −COOH might be the reason for the fast adsorption of MB.

The effect of different temperatures (298, 308, 318, 328, and 338 K) on the adsorption of MB by C–CMP was investigated. As the temperature changed from 298 to 338 K, the adsorption amount increased from 207.28 mg g^−1^ to 272.13 mg g^−1^ (Figure S6). The increase in temperature not only accelerates the diffusion rate, but also increases the activity of adsorption sites to bind MB molecules [33,34].

In order to study the thermodynamics of MB adsorption, the thermodynamic parameters were calculated as follows [31]:

(5) $$K_{C}=\frac{Q_{e}}{C_{e}}$$

(6) $${lnK}_{C}=\frac{{\Delta S}^{0}}{R}–\frac{{\Delta H}^{0}}{RT}$$

(7) $${\Delta G}^{0}{\;=-RTlnK}_{C}$$

where *R* (8.314 J·mol^−1^·K^−1^) is the ideal gas constant, *K_c_* is the adsorption equilibrium constant, and Δ*H*^0^ (kJ·mol^−1^), Δ*S*^0^ (kJ·mol^−1^·K^−1^) and Δ*G*^0^ (kJ·mol^−1^) represent the standard enthalpy, entropy, and the standard Gibbs free energy, respectively, and Δ*G* is calculated from Equation (7). The effect of temperature on the thermodynamics of Brownian motion molecules suggests that adsorption is an endothermic process (Figure 4e) [35]. According to the thermodynamic parameters in Table S4, Δ*G*^0^ < 0 indicated that the adsorption is spontaneous, while Δ*S*^0^ > 0 indicates that the adsorption of MB dye by C–CMP foam is a process of increasing entropy.

An excellent adsorbent should not only have high adsorption capacity, but also have good reusability. The adsorption–desorption experiment was conducted by three cycles, and 0.1 mol L^−1^ HCl was used to realize the desorption of MB on the surface of C–CMP. Figure 4f shows that the MB removal efficiency by C–CMP foam did not decrease significantly, and it was still above 80% after three cycles, confirming good recyclability of C–CMP composite foam. The slightly decreased efficiency may be ascribed to the strong interaction of MB with C–CMP and the residual dye that was adsorbed from the previous cycles.

### 2.3. Adsorption Mechanism

**Figure 5:**
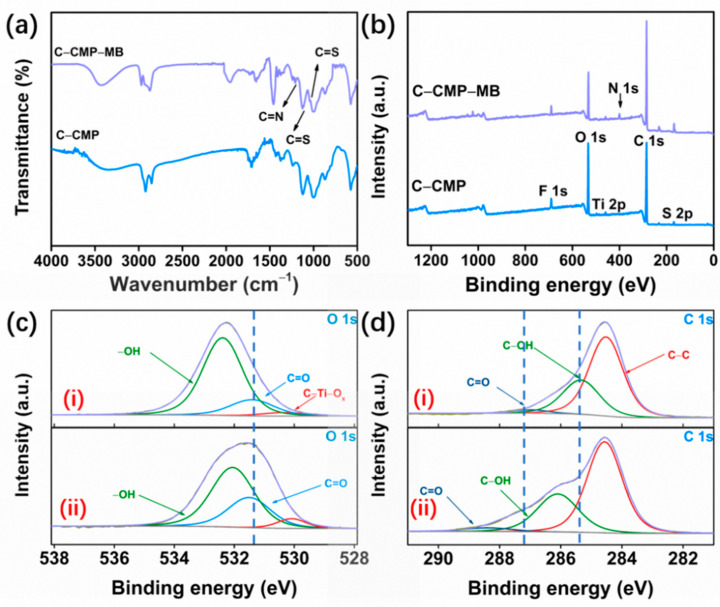
(**a**) FT–IR spectra of the fresh C–CMP and C–CMP after MB removal (C–CMP–MB). (**b**) X-ray photoelectron spectra (XPS) of C–CMP and C–CMP–MB. (**c**) O 1s scans of C–CMP (**i**) and C–CMP–MB (ii). (**d**) C 1s scans of C–CMP (**i**) and C–CMP–MB (**ii**).

The effect of the strong interaction of MB molecules with C–CMP was studied by FT–IR, EDX and X-ray photoelectron spectroscopy (XPS). As displayed in Figure 5a, additional peaks can be observed at 1209, 1116/1042 cm^−1^, which were assigned to the C=N, C=S stretching vibration of MB after the MB adsorption by C–CMP foam (C–CMP–MB) [36]. Besides, the EDX results (Table S1) show that after C–CMP adsorbs MB molecules, the N element (0.60 wt%) in C–CMP–MB begins to appear, and the contents of the whole organic elements (97.04 wt%) are slightly increased compared with C–CMP (94.00 wt%), which confirms that C–CMP can have an effective removal effect on MB dye.

Figure 5b shows the difference in the XPS spectra of C–CMP before and after adsorption of MB due to the presence of the N element in C–CMP–MB. The peaks of high–resolution XPS spectra of O 1s located at 532.4 and 531.4 eV in C–CMP were assigned to the −OH and C=O, respectively (Figure 5c(i)). After MB adsorption onto C–CMP, the intensity decreases in the −OH peak and C=O peak shifted to higher electron energy, indicating the binding effect between C–CMP and MB molecules (Figure 5c(ii)). The three peaks centered at 287.0 eV (C=O), 285.4 eV (C–OH) and 284.6 eV (C–C) were used to fit the high–resolution C 1s spectrum (Figure 5d(i)). It can be seen that the binding energy of C=O and C–OH peaks also exhibit a positive shift after the MB adsorption process (Figure 5d(ii)) [37]. We speculate that the −COOH and −OH functional groups on the surface of C–CMP are easily ionized, resulting in COO^−^ and O^−^, which can attract MB (S^+^) by electrostatic interaction [36,38]. Besides, the pH value of the solution was measured after adsorption of MB. The reduced pH indicates that the proton exchange to release H^+^ ions occurred during the process. Therefore, −OH and −COOH play a huge role in the interaction between C–CMP and MB molecules.

## 3. Materials and Methods

### 3.1. Materials

Ti_3_AlC_2_ (purity > 99.99%, 400 mesh) was purchased from Jilin 11 Technology Co., Ltd., Hangzhou, China. Sodium dodecyl sulfate (≥98%), glutaraldehyde (50% in H_2_O), formaldehyde solution (AR) and methylene blue (MB) (≥98%) were supplied by Aladdin Chemistry Co., Ltd., Shanghai, China. Polyvinyl alcohol (PVA) (M_w_ = 85,000–124,000, 87–89%) was procured from Sigma–Aldrich Chemical Co., Ltd., Shanghai, China. Cellulose nanocrystal (CNC) (99.6%) was provided by Guilin Qihong Technology Co., Ltd., Guilin, China. All chemical reagents were used without further purification in all experiments.

### 3.2. Synthesis of Ti_3_C_2_T_x_ MXene

Ti_3_C_2_T_x_ MXene was obtained by using the HF acid method to etch away aluminum from the MAX phase (Ti_3_AlC_2_). In a typical reaction, 1 g Ti_3_AlC_2_ was added to 20 mL of 40% HF solution. The reaction was vigorously stirred and carried out at room temperature for 24 h. Then, the resulting suspension was treated by alternatively centrifuging, redispersing and sonicating until the pH = 6.0. The resultant MXene powder was obtained by freeze–drying at −80 °C for 24 h.

### 3.3. Synthesis of CMP Composite Foam

PVA (5 g) was firstly dissolved into CNC aqueous solution (1 wt%, 45 mL) and then it was sonicated to remove bubbles. After that, an appropriate amount of 5 mg mL^−1^ MXene aqueous dispersion, 6.5 g sodium dodecyl sulfate, 1 mL formaldehyde solution (96 wt%), and 5 mL diluted H_2_SO_4_ solution (20 wt%) were added to the solution in sequence. It was stirred at 200 rpm for 5 h to make the solution uniform and foamed for 2 min with a vortex shaker. CMP composite foam was obtained after the foamed solution was poured into a polytetrafluoroethylene mold and dried and shaped in a vacuum oven at 60 °C for 24 h. PVA, CNC/PVA, and MXene/PVA foams were prepared in the same manners as the control experiments.

### 3.4. Synthesis of C–CMP Composite Foam 

C–CMP composite foam was prepared using CMP foam cross–linked with a solution (15 wt%) of GLA and acetone (pH = 2) (25 °C) for 6 h, then the foam was washed several times with ethanol, and dried at room temperature.

### 3.5. Characterization 

X-ray diffraction (XRD) patterns were performed on a D8 ADVANCE 03030502X’Pert diffractometer (Bruker, Germany) with Cu Kα radiation (λ = 1.5406 Å). Fourier transform infrared (FTIR) spectra were recorded using a Nexus 670 spectrometer (Thermo Nicolet, Thermo Fisher Scientific, Waltham, MA, USA) in the range of 4000–400 cm^−1^. The contents of C, H, O, N and S of the samples were investigated using Elemental Analyzer (Elementar, vario EL III). XPS data was acquired on the ESCALAB Xi+ (Thermo Fisher Scientific, Waltham, MA, USA) X-ray photoelectron spectrometer. TGA of the composite foams was performed on SDT–Q600 (TA Instruments, New Castle, DE, USA) from 40 °C to 600 °C at a heating rate of 10 °C/min in N_2_ atmosphere. SEM (Hitachi Regulus 8220, Hitachi High-Technologies Corporation, Tokyo, Japan) with energy dispersive spectrum (EDS) was employed to show the surface morphology and elemental weight percentage of the adsorbent. Splines of the foams were stretched at a speed of 5 mm per minute under room temperature by a computer–controlled electronic universal testing machine (Hensgrand, WDW–02, Jinan, China). Contents of MB and other dyes were determined by an ultraviolet–visible spectrophotometer (UV-2600, Shimadzu, Kyoto, Japan) at 664 nm.

### 3.6. Batch Adsorption Experiments

The influence of pH value, initial MB concentration, MB contact time and temperature on the adsorption capacity of composite foams were studied by batch experiments. An appropriate amount of MB powder was dissolved in DI water to prepare 500 mg L^−1^ MB stock solution, which was kept away from light. In subsequent experiments, 10 mg of adsorbent was added to 20 mL of MB solution in a beaker. The pH value was adjusted to 6.0 with 0.1 M NaOH or 0.1 M HCl. The solution was filtered through a PES membrane after it was stirred at room temperature for 12 h. The filtrate was then diluted to determine MB concentration using the ultraviolet–visible spectrophotometer. The adsorption capacity *Q_e_* (mg·g^−1^) was calculated as *Q_e_* = (*C*_0_–*C_e_*) *V*/*m*, where *C*_0_ and *C_e_* are the initial and final equilibrium concentrations of MB, *V* and *m* are the volume of the solution and mass of the adsorbent in the tests. Unless otherwise specified, the detailed information for all adsorption conditions was: *m*/*V* = 0.5 mg mL^−1^, initial concentration of MB: 250 mg·L^−1^, pH = 6.0, t = 12 h, T = 273 K.

## 4. Conclusions

Macroporous cellulose nanocrystals (CNC)/MXene/polyvinyl alcohol (PVA) (C–CMP) foam (C–CMP) with excellent mechanical and adsorption properties was prepared using a simple and green foaming method. The adsorption experiments on contact time and initial MB concentration showed that MB could be rapidly adsorbed and reached equilibrium in ~3 h, with a maximum adsorption capacity of about 239.92 mg·g^−1^. Compared with the anionic dye MO (45.25 mg g^−1^), C–CMP was more suitable for the removal of cationic dyes by adsorption. Moreover, the MB adsorption over C–CMP composite foam followed the Langmuir isotherm and pseudo–second–order kinetic models, corresponding to monolayer adsorption and chemisorption. From the thermodynamic analysis, the adsorption was spontaneous (Δ*G*^0^ < 0) and endothermic (Δ*H*^0^ = 10.596 kJ mol^−1^). Furthermore, the adsorption mechanism of C–CMP was dominated by electrostatic attraction. Considering the simple preparation process and good adsorption performance of C–CMP foam, it is considered to be an ideal candidate for the removal of cationic pollutants in wastewater.

## Figures and Tables

**Table 1:** Tensile data of different samples.

Samples	Elongation at Break (%)	Tensile Strength (Mpa)
PVA	43.96 ± 4.80	0.24 ± 0.02
PVA/CNC	50.96 ± 4.24	1.88 ± 0.07
PVA/MXene	70.44 ± 6.12	2.95 ± 0.21
CMP	99.9 ± 10.70	1.71 ± 0.18
C–CMP	75.1 ± 8.04	2.03 ± 0.12

## Data Availability

The data presented in this study are available in the Supplementary Materials.

## Supplementary Materials

The following supporting information can be downloaded at: www.mdpi.com/article/10.3390/molecules27134243/s1, Figure S1: SEM image of Ti_3_C_2_T_x_ MXene; Figure S2: MB adsorption from aqueous solution onto C–CMP as a function of pH value. Initial concentration of MB: 250 mg L^−1^. t = 12 h. T = 273 K; Figure S3: Equilibrium adsorption capacity of MB dye at various initial concentration. pH = 6.0. t = 12 h. T = 273 K; Figure S4: Equilibrium adsorption capacity of MO dye at various initial concentration. pH = 6.0. t = 12 h. T = 273 K; Figure S5: Effect of contact time for MB removal over C–CMP. Initial concentration of MB: 250 mg L^−1^. pH = 6.0. T = 273 K; Figure S6: Effect of temperature for MB removal over C–CMP. Initial concentration of MB: 250 mg L^−1^. pH = 6.0. t = 12 h; Table S1: EDX of CMP, C–CMP and C–CMP–MB; Table S2: Parameters of the Langmuir and Freundlich models for the adsorption of MB by C–CMP; Table S3: Kinetic parameters of the pseudo–first–order and pseudo–second order models for MB adsorption by C–CMP; Table S4: Thermodynamic parameters of MB removal on C–CMP.

## Sample Availability

Samples of the compounds are not available from the authors.
