# Supplementary material for: One-Pot Synthesis of Cellulose/MXene/PVA Foam for Efficient Methylene Blue Removal
Source: Molecules. 2022 Jun 30;27(13):4243. doi: 10.3390/molecules27134243 (PMC9268378; doi:10.3390/molecules27134243)
Supplement: Supplementary file 1 [file molecules-27-04243-s001.zip › molecules-1794968-supplementary.pdf]

Supplementary Materials

One-Pot Synthesis of Cellulose/MXene/PVA Foam for Efficient Methylene Blue Removal

Weisong Zhao, Hong Chi *, Shiyun Zhang, Xue Zhang and Tianduo Li *

Shandong Provincial Key Laboratory of Molecular Engineering, School of Chemistry of Chemical Engineering, Qilu University of Technology (Shandong Academy of Sciences), Jinan 250353, China; wsmail2022@163.com (W.Z.); whitesnow0613@163.com (S.Z.); chihqut@163.com (X.Z.)

* *Correspondence: chihong@qlu.edu.cn (H.C.); litianduo@163.com (T.L.)*

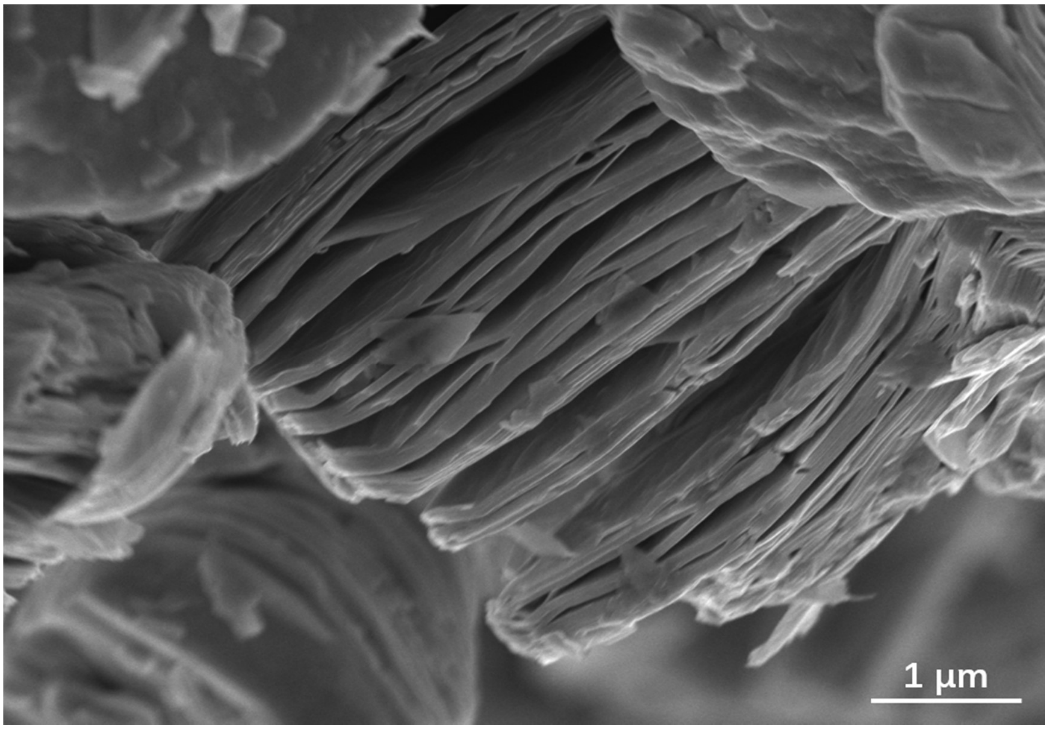

Figure S1. SEM image of $Ti_3C_2T_x$ MXene.

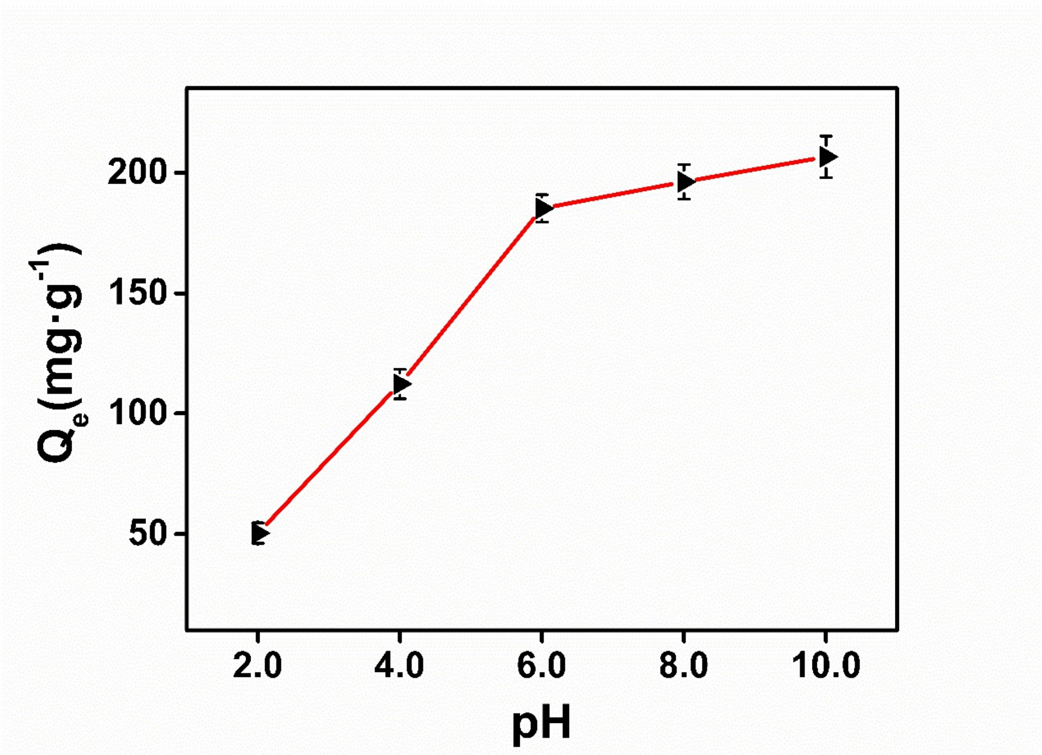

Figure S2. MB adsorption from aqueous solution onto C-CMP as a function of pH value. Initial concentration of MB: 250 mg L^{-1}. T = 12 h. T = 273 K.

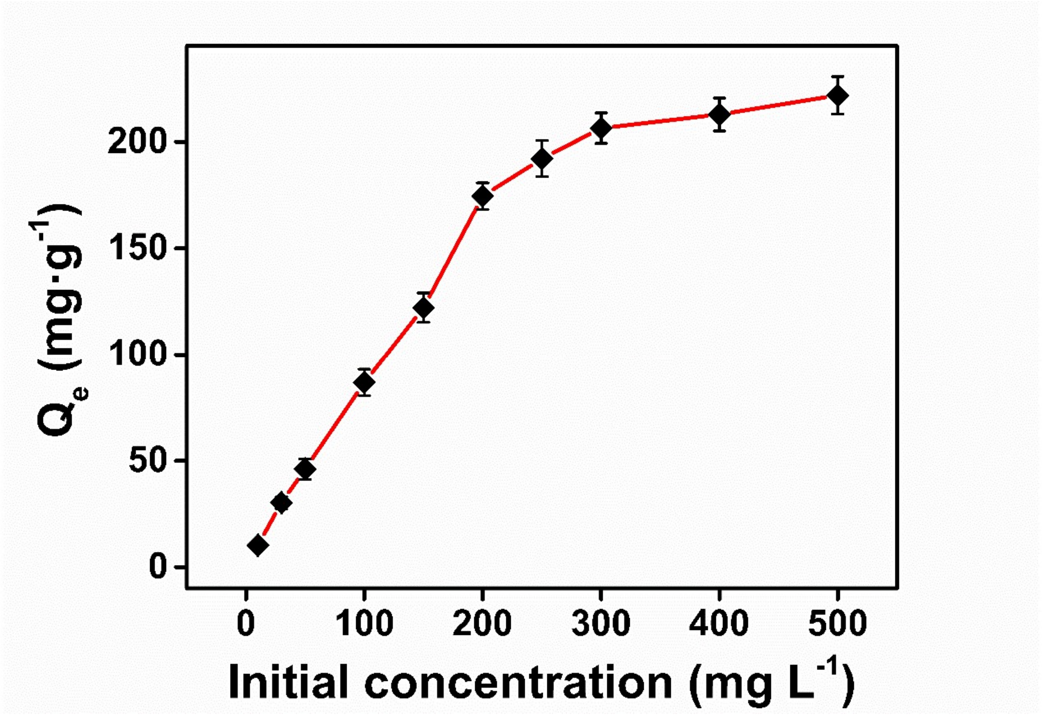

Figure S3. Equilibrium adsorption capacity of MB dye at various initial concentration. pH = 6.0. t = 12 h. T = 273 K.

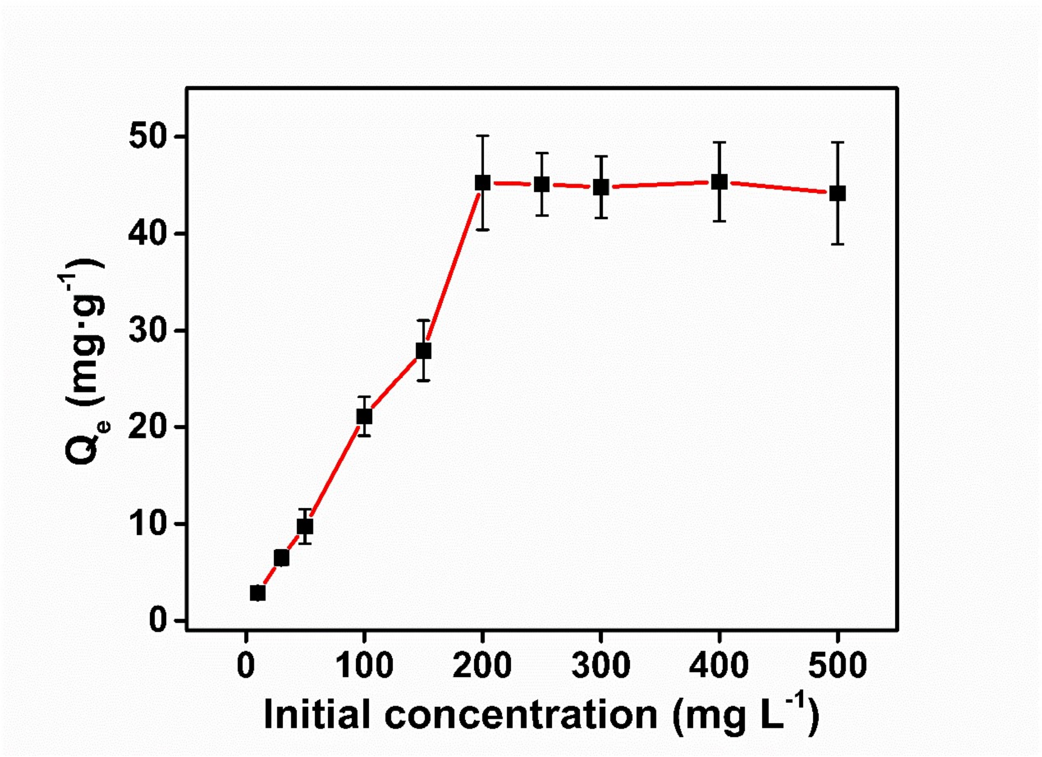

Figure S4. Equilibrium adsorption capacity of MO dye at various initial concentration. pH = 6.0. t = 12 h. T = 273 K.

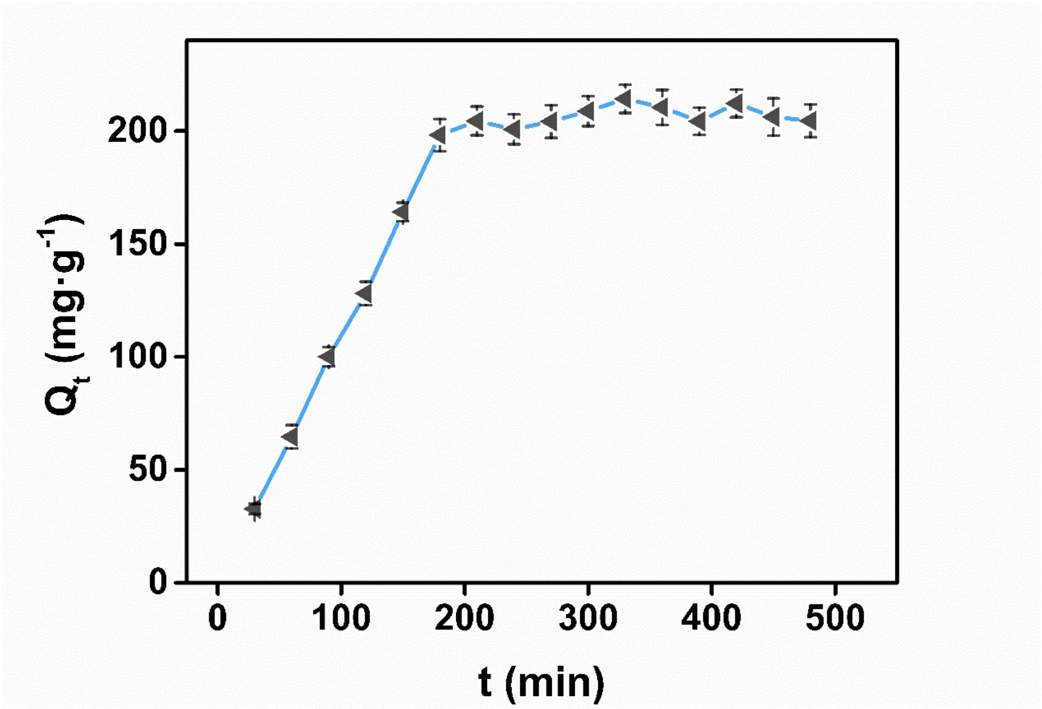

Figure S5. Effect of contact time for MB removal over C-CMP. Initial concentration of MB: 250 mg L^{-1}. pH=6.0. T=273 K.

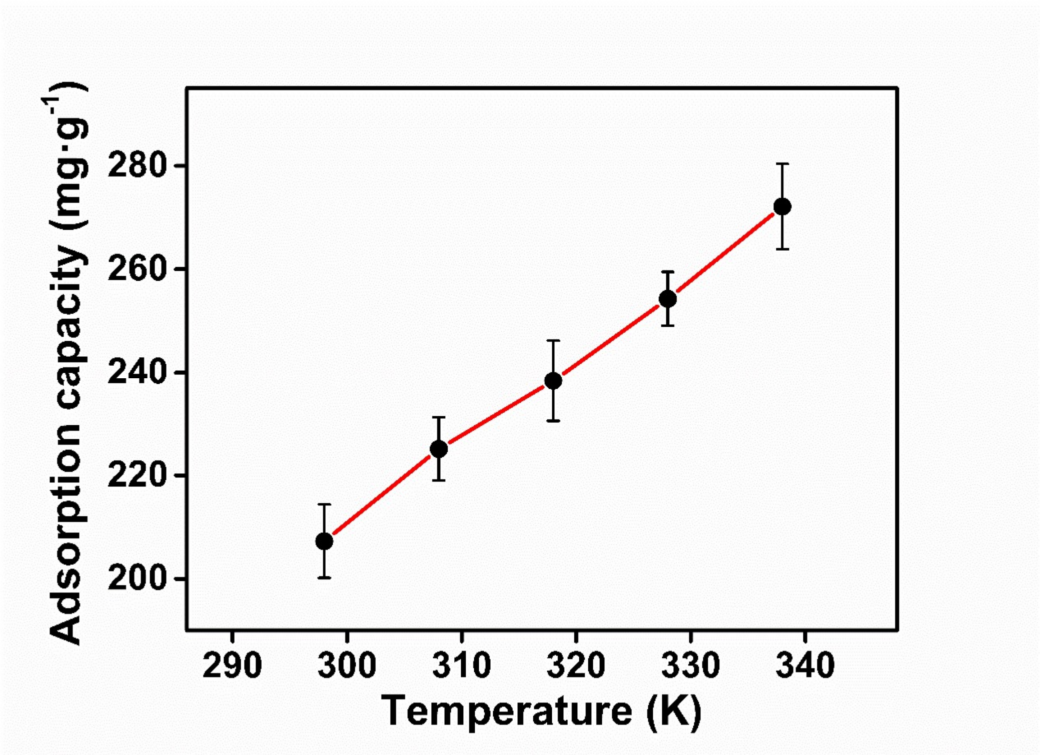

Figure S6. Effect of temperature for MB removal over C-CMP. Initial concentration of MB: 250 mg L^{-1}. pH = 6.0. t = 12 h.

Table S1. EDX results of CMP, C-CMP and C-CMP-MB.

Sample	C (wt%)	O (wt%)	S (wt%)	H (wt%)	N (wt%)	Total ratio (wt%)
CMP	48.23	35.23	1.12	7.92	0.00	92.50
C-CMP	52.91	33.36	0.85	6.90	0.00	94.00
C-CMP-MB	53.20	34.75	1.23	7.26	0.60	97.04

Table S2. Parameters of the Langmuir and Freundlich models for the adsorption of MB by C-CMP.

Adsorbate	Langmuir parameters			Freundlich parameters		
	K_L (L/mg)	q_m $(mg\ g^{-1})$	R^2	K_F (L/mg)	n	R^2
MB	0.00532	239.92	0.97284	3.31945	1.33207	0.92385

Table S3. Kinetic parameters of the pseudo-first-order and pseudo-second order models for MB adsorption by C-CMP.

Initial concentration (mg L^{-1})	Pseudo-first-order model			Pseudo-second-order model		
	k_1 (min^{-1})	q_e $(mg\ g^{-1})$	R^2	$k_2\ (g\ mg^{-1}\ min^{-1})$	q_e $(mg\ g^{-1})$	R^2
MB-250	0.0070	231.49	0.94757	0.00017	221.24	0.97159

Table S4. Thermodynamic parameters of MB removal on C-CMP.

**T (K)**	***ΔH⁰ (kJ·mol⁻¹)***	***ΔS⁰ (kJ·mol⁻¹·K⁻¹)***	***ΔG⁰ (kJ·mol⁻¹)***
298	-	-	-0.862
308	-	-	-1.263
318	10.596	0.0385	-1.586
328	-	-	-1.985
338	-	-	-2.445
